# The Relationship between COVID-19 and Innate Immunity in Children: A Review

**DOI:** 10.3390/children8040266

**Published:** 2021-03-30

**Authors:** Piero Valentini, Giorgio Sodero, Danilo Buonsenso

**Affiliations:** 1 Istituto di Pediatria, Università Cattolica del Sacro Cuore, 00168 Rome, Italy; piero.valentini@policlinicogemelli.it (P.V.); giorgio.sodero@hotmail.it (G.S.); 2 Department of Woman and Child Health and Public Health, Fondazione Policlinico Universitario A. Gemelli IRCCS, 00168 Rome, Italy; 3 Global Health Research Institute, Istituto di Igiene, Università Cattolica del Sacro Cuore, 00168 Rome, Italy; 4 Dipartimento di Scienze Biotecnologiche di Base, Cliniche Intensivologiche e Perioperatorie, Università Cattolica del Sacro Cuore, 00168 Rome, Italy

**Keywords:** COVID-19, children, coronavirus, innate immunity, SARS-CoV-2, pandemic, MIC-S

## Abstract

Severe acute respiratory syndrome coronavirus 2 (SARS-CoV-2) is the virus responsible for the pandemic viral pneumonia that was first identified in Wuhan, China, in December 2019, and has since rapidly spread around the world. The number of COVID-19 cases recorded in pediatric age is around 1% of the total. The immunological mechanisms that lead to a lower susceptibility or severity of pediatric patients are not entirely clear. At the same time, the immune dysregulation found in those children who developed the multisystem inflammatory syndrome (MIC-S) is not yet fully understood. The aim of this review is to analyze the possible influence of children’s innate immune systems, considering the risk of contracting the virus, spreading it, and developing symptomatic disease or complications related to infection.

## 1. Introduction

Severe acute respiratory syndrome coronavirus 2 (SARS-CoV-2) is the virus responsible for the pandemic viral pneumonia (known as COVID-19) that was first identified in Wuhan, China, in December 2019, and rapidly spread around the world [1]. Children account for a minority of the total patients [2]. During the pandemic, the number of sick children presenting to hospital has decreased significantly, mainly due to lockdown, a reduction in the circulation of infectious diseases, and fear of hospitals [3]. Children have also experienced a change in their social and family life habits, mainly due to school closures, which have been implemented in the majority of countries worldwide in efforts to contain the pandemic [4]. Even today, the immunological mechanisms that lead to a lower susceptibility or severity in pediatric patients are not entirely clear. Similarly, the immune dysregulation found in those children who developed the multisystem inflammatory syndrome (MIC-S) is not yet fully understood.

The aim of this review is to analyze the possible influence of children’s innate immune systems regarding the risk of contracting the virus, spreading it, and developing symptomatic disease or complications related to the infection.

## 2. COVID-19 and Children

The clinical presentation of SARS-CoV-2 infection in children is mild in most cases [5], although disease progression toward more severe forms is possible. The number of cases recorded in the pediatric age is around 1% of the total. Because of the usually mild clinical presentation, this percentage mainly refers to the few symptomatic patients or tests performed for contact tracing, and could therefore be underestimated [6]. There are several theories about the differences of COVID-19 severity in children and adults, based on the type of immune response, different levels of the expression of angiotensin-converting enzyme (ACE) 2 receptor (necessary for viral adhesion and replication), or the competitive action of other respiratory viruses that colonize the nasopharyngeal mucosa in the pediatric age [7]. However, there is currently insufficient evidence to confirm these hypotheses. It has been shown that children with COVID-19 have a stronger innate immune response in the nasopharyngeal mucosa than adult patients, which is associated with a greater expression of several cytokines (such as IL-17A, interferon (IFN) gamma, IFN-alpha2, IL-1b, IL-8, and IP10) [8], which could partially explain the lower susceptibility to infection. The CONFIDENCE research group [9], analyzing 170 Italian children with a confirmed diagnosis of COVID-19 in 17 different Italian pediatric emergency departments, showed that the most frequent clinical presentation was ill appearance (12%), fever (48%), cough, (73.43%), problems with feeding (42.35%), and rhinorrhea (34.20%); less common symptoms were apnea, cyanosis, headache, and dehydration. Although the SARS-CoV-2 disease mainly affects the respiratory system, gastrointestinal manifestations are also possible (mainly represented by diarrhea and abdominal pain), which may appear at the onset of the disease or later. [5] Similar data on clinical presentation have been reported in other countries [10,11]. In addition to the clinical impact, this disease has had a psychological influence on children: the lack social interactions has increased the incidence of anxiety, lethargy, and depression [12]. Furthermore, the closure of schools in many countries of the world, such as in Italy, has contributed to the exacerbation of these problems [13].

## 3. Immunological Response to Infection

### 3.1. Susceptibility

The immune system plays a key role in the body’s response to this new coronavirus. Despite the doubts about the pathogenetic mechanisms, it is evident that innate immunity influences the onset of symptoms and the severity of COVID-19, while adaptive immunity is mainly involved in viral clearance [14]. The body responds to SARS-CoV-2 infection as it does to other respiratory viruses, counteracting the infection on multiple levels. In children, the adaptive immunity is not yet completely developed; unlike during adulthood, adaptive immunity may also be reduced in the context of viral diseases. Therefore, innate mechanisms could play an important role in the response to these infections [15].

Infection is usually acquired horizontally through the inhalation of droplet particles; the skin and mucous membranes represent the first barrier against external pathogens. Respiratory viruses generally enter the body through the nasal, oral, or conjunctival mucosa, and are mainly hindered by the IgA present in secretions; the virus-specific IgA-mediated immune response of the mucous membranes of COVID-19 patients appear to be detected in the majority of patients, and may be more effective and lasting than the IgM-mediated response [14]. There is an inverse relationship between amounts of SARS-CoV-2-specific antibodies (IgG and IgA) in the nasopharyngeal mucosa and IL-18 levels, with no differences between adults and children. In addition, higher levels of this cytokine are associated with more severe forms of the disease. It can be hypothesized that the active form of IL-18, interacting with the interferon pathway, negatively modulates the adaptive immunological response of the organism [8].

The structure of virions is composed of the membrane (M), envelope (E), nucleocapsid (N), and spike (S) proteins, which interact with the angiotensin-converting enzyme 2 (ACE2) [16].

It has recently been shown that the expression of ACE2 on the cells of the respiratory mucosa, essential for the penetration of SARS-Cov-2, varies over the years of growth [8], reaching higher levels in adulthood. Children less than ten years old have the lowest ranges of expression. These considerations could, at least partially, explain their lower susceptibility to infection, without, however, justifying the high frequency of asymptomatic positive children. Moreover, it is yet unclear whether the new variants found in the United Kingdom and South Africa have a major affinity with receptors more represented in children.

The lower incidence in children could also be explained by anatomical mechanisms, such as the greater resistance of the upper airways in children (aerosol particles are deposited more in the tracheobronchial tree than in the alveoli), or by the greater regenerative capacity of the pediatric lungs. Other non-COVID-19 coronaviruses are detectable in respiratory secretions in a large percentage of healthy children [17], and it is unclear whether the presence of multiple viruses in the airways can exert a biological competition mechanism.

A correlation between Human leukocyte antigen (HLA) alleles and virus susceptibility has not yet been proven, although a higher frequency of DRB1*15:01 and DQB1*06: 02 has been observed in patients with severe disease [18]. Although little is known about Sars-Cov-2, the similarity with Sars-Cov has led to insights into the pathogenetic mechanisms, such as the role of IL-6 [19], which is high in many inflammatory processes but generally lower in children than in affected adults. The increase in procalcitonin and C-reactive protein may not be present in some pediatric cases, although it is useful to monitor the degree of inflammation in order to identify early disease progression or complications such as bacterial pneumonia.

The vertical transmission of Sars-Cov-2, transplacental or transcervical, has been reported in sporadic cases of maternal–fetal transmission reported in the literature [20], which is associated with placental inflammation, neonatal viremia, and the possibility of cerebral vasculitis. Despite the often-favorable neonatal outcome, hospitalized mothers infected with coronavirus infections are at increased risk of adverse obstetric outcomes such as preterm delivery, preeclampsia, cesarean delivery, and perinatal death [21].

### 3.2. Complement System and Cytokines

Sars-Cov-2 infection can activate the complement through the lectin pathway [22] and generates an inflammatory response through mediators such as C3a and C5a, contributing to cell recruitment and to the activation of inflammatory cells [23]. In addition, neutrophil levels can increase during COVID-19, with a possible concordance between neutrophilia and pulmonary radiological abnormalities [24]. An increase in ferritin, transaminases, and various cytokines (including IL-6, IL-10, G-CSF, and others) produced by macrophages may occur during infection, especially in severe pediatric and adult patients [25]. High levels of lactate dehydrogenase (LDH), a not-specific marker usually associated with cardiopulmonary problems and inflammation, are more commonly found in children than adults, despite the minor severity of the disease [26]. SARS-CoV-2 alters the type I IFN (interferon) system, with over ten antiviral proteins, such as NSP16, which suppresses global mRNA splicing and decreases the recognition of viral RNA by intracellular receptors; NSP1, which leads to the global inhibition of mRNA translation; and NSP8 and NSP9, which interfere with protein transfer to the cell membrane [27]. All these mechanisms independently lead to a reduction of type I IFN production, causing impaired communication between the innate and adaptive immunity, and an altered communication between proinflammatory and antiviral activity. Some studies have also shown significantly higher levels of IFN gamma and IFN alpha2 in the nasopharyngeal fluid of pediatric patients, also showing an increased blood response compared with adult patients [8]. These findings may partly explain the improved immune efficiency of children against Sars-CoV-2.

### 3.3. Entering the Target Cells

The activity of neutrophils and the levels of proinflammatory cytokines are correlated to the age of the patient, being lower in children, and contribute to the severity of respiratory manifestations [28]. After entering the target cells, the virus is tracked by pattern recognition receptors such as Toll-like receptors 3, 7, 8, and 9 and viral-infection sensors RIG-I and MDA5; different Tool Like Receptor (TLRs) trigger the transcription of the NLR family pyrin domain containing 3 (NLRP3) gene, and contribute to the activation of the inflammasome complexes, inducing the production of key proinflammatory cytokines IL-1β and IL-18 as well as the caspase-1 activation [29]. This mechanism does not appear to be different between children and adults. The virus may also escape the immune response with the inhibition of the type I IFN pathway through direct or indirect mechanisms (such as functional inhibition of macrophages, dendritic cells, and Natural Killer (NK) mediated by viral TLR ligands that could induce unwanted polarization of the cell membrane through different molecular mechanisms). This would have deleterious consequences not only on the antiviral activity of the innate cells themselves, but also on the downstream adaptive responses [30]. These set of mechanisms can lead to a reduction in the efficiency of innate immunity, as well as a failure to activate acquired and adaptive immunity. Although the response to Sars-CoV-2 infection is influenced by immune status, immunosuppressed patients appear to have the same susceptibility as the general population for contracting the virus and transmitting it, without a greater risk of severe forms of COVID-19 [29].

### 3.4. Mast Cells

Mast cells are another component of innate immunity that can counteract infectious processes (bacterial, fungal, or viral) or trigger various proinflammatory reactions [31]. They can participate in the recognition of viruses through different mechanisms (such as the expression of TLR-3), and be directly infected by Sars-CoV-2, as they express the ACE2 receptor and other serine proteases necessary for infection [32]. The response to viral infection results in the release of several proinflammatory molecules, including histamine, tryptase, IL-1β, CCL2, IL-6, GM-CSF, and TNF-α, which are implicated in COVID-19 and appear to have relationship with pulmonary damage and fibrosis [33]. The different mast cell activation in pediatric SARS-CoV-2 has not yet been investigated, although we do not expect a significant difference in mast cell action. The use of H2 receptor antagonists, counteracting the effect of histamine and also having a potential immunomodulatory effect, has been hypothesized as a possible therapy for COVID-19, interfering with the progression towards severe forms [34].

### 3.5. Lymphocites

An increase or decrease in neutrophils, NK, and other white blood cells can occur in children with COVID-19, although most pediatric cases have normal white blood cell counts with normal lymphocytes levels (Table 1) [35]. When lymphopenia is the major laboratory finding, all lymphocyte subgroups are decreased, along with increased exhaustion levels and reduced functional diversity [36]. Although a decrease in CD4+ T cells was common in adult patients with severe and moderate COVID-19, this finding was rarely seen in children. At birth, there is a greater secretion capacity of IL-10, a cytokine capable of inhibiting inflammatory processes, while in the following years, a greater balance between T helper (Th) 1/Th2/Th17 lymphocytes develops; this finding, described as the immunosenescence phenomenon, helps to explain the different immunological response in adults compared with pediatric patients [37,38].

In adults, the severity of the respiratory pathology would appear to be associated with a reduction of plasma lymphocytes; in pediatric patients with lymphopenia, this association is less evident, considering that the causes of lymphopenia in very young children can be multiple or physiological, and not necessarily associated with the ongoing viral infection [35].

### 3.6. Eosinophils

Eosinophils also play an important inflammatory role, especially in the upper airways; in COVID-19, there is evidence of low eosinophil levels associated with lymphopenia [39] because of multifactorial causes, related to a reduction in bone marrow production, an increase in apoptosis, and a reduction in the expression of chemokines and cell adhesion factors. Eosinophil counts would normalize with the patient’s clinical improvement, suggesting that these cells could play a protective role towards this infection. Although the evidence is still limited, eosinophils may play a protective role during SARS-CoV-2 infection, and eosinopenia may serve as a prognostic indicator for more severe COVID-19 [39]. It is not yet known whether the diseases associated with hypereosinophilia (such as, allergic diseases) have a protective relationship or not towards Sars-CoV-2. For example, pediatric asthma patients, who normally have higher eosinophil levels than the general population, do not appear to have a greater susceptibility to Sars-Cov-2 [40], and, unlike other common childhood respiratory viruses, this pathogen does not cause disease flare-ups. Asthma and, in general, other allergic-based diseases would therefore not seem to be a risk factor for this new infection [41], with some cases even showing a protective effect. It is also possible to hypothesize that SARS-CoV-2 may not induce bronchial hyperreactivity and other pathophysiological mechanisms involved in asthma exacerbations [40].

## 4. Protective Immunity

Both humoral and cell-mediated immunity are necessary to elicit protective immunity against Sars-CoV-2 reinfection [42]. Immunity acquired following primary infection may protect from subsequent exposure to the virus [43], even if in the literature there are some cases of COVID-19 patients with symptoms relapses, in association with the positivity of the nasopharyngeal molecular swab. It is important to establish the difference between reinfection, relapse, and PCR new positivity [42], because, as the duration of immunity against Sars-CoV-2 is still unknown, it is difficult to establish whether some recidivated cases described in the literature are an exception. We know that in pediatric cases of sustained positivity and reinfection described in literature, the mean duration of virus shedding in respiratory secretion was 34 days [44]. The mechanism of antibody-dependent enhancement (ADE), the production of non-neutralizing antibodies to the virus, has been proposed to describe the severity of some cases of COVID-19 and the appearance of a new infection, although it has not yet been demonstrated [29]. The duration of protection from the virus (after acquiring the disease or after vaccination) and any differences in permanent or transient immunization between children and adults are not yet fully known.

## 5. Trained Immunity: The Role of Bacillus Calmette–Guérin (BCG) Vaccination

Trained immunity is the functional re-programming of innate immune cells to a more activated state following initial antigen stimulation through metabolic and epigenetic changes, which can affect the progenitor cells of myeloid and monocytic cell lines, as well as local cells [45]. In addition to the main effect, vaccinations (like infections) are capable of generating nonspecific effects through cross-reactions between vaccine antigens and antigens from unrelated pathogens. These modifications subsequently regulate the production of cytokines and cellular metabolism in a bidirectional way [46]. Several vaccinations with live attenuated microorganisms are capable of inducing this “trained immunity”, which can cross-protect against other infections, such as mycobacterium tuberculosis or various respiratory viruses (respiratory syncytial virus or influenza A and B) [47]. In particular, BCG has shown that the innate immune system can develop a trained immunity through the epigenetic reprogramming of different innate immune cell types [48], which can help the body to respond to viruses. It has been suggested that countries that include Bacillus Calmette–Guérin in their vaccination calendar have fewer confirmed cases and a lower mortality in Sars-Cov-2 patients than in countries without [49], showing a possible inverse correlation between BCG and COVID-19 incidence and mortality. BCG vaccination administered in the neonatal period protects against non-tuberculous infectious diseases during the first weeks of life, as well as having an already proven anti-tuberculosis efficacy; the non-specific effects of BCG for infectious morbidity and all-cause mortality suggest that the administration of BCG on the day of birth should be a priority in areas with a high prevalence of infectious diseases and could also play a role against Sars-CoV-2 infection [50]. However, there are several confounding factors that are not considered in this correlation [51], like comorbidity or different rates of progress for the pandemic among the various populations considered. Moreover, adult patients, in most cases, have received the same vaccines given to children during their lifetime, and this consideration can lead to the exclusion of this theory or to consider its role only in acute infection. Therefore, future studies should focus on the true extent of the protection offered by trained immunity.

## 6. Immune Dysregulation and Sars-CoV-2 Infection

A minority of children who contract the infection may develop an intense inflammatory response caused by the cytokine storm induced by Sars-CoV-2, called “multisystem inflammatory syndrome in children and adolescents temporally related to COVID19”, also known as MIS-C [52]. Initially described as a variant of Kawasaki disease, due to frequent cardiac impairment, it has been associated with several other inflammatory diseases such as acute rheumatic fever (ARF) or toxic shock syndrome (TSS) [53]. MIC-S is defined by the presence of more than 3 days of fever in children aged up to 19 years, diagnosed with COVID19 infection or confirmed contact with a positive case, increased inflammation indices in the absence of other microbial cause of inflammation, and at least two clinical signs (skin rash or muco-cutaneous inflammation signs, hypotension or shock, cardiac involvement, coagulopathy, and gastrointestinal symptoms) [54]. In addition to the alterations previously described, this syndrome may cause thrombocytopenia, elevation of the D-dimer, prolonged prothrombin time, and Blood Natriuretic Peptide (BNP) levels correlated inversely with cardiac ejection fraction [55], worsening the prognosis of affected children. Although the immune response, especially the innate immunity, is essential for controlling the early stages of infection, severe forms of MIC-S require modulation to prevent the development of complications [56]. Despite massive systemic involvement, cases of acute respiratory failure are much rarer than in the adult population, even in severe forms of MIC-S. Among the various theories, we know that there is a distinct antibody response in children with MIS-C compared with adults with severe COVID-19, because MIS-C predominantly generated IsgG antibodies specific for the spike (S) protein but not for the nucleocapsid (N) protein, while the adult COVID-19 cohorts had anti-S IgG, IgM, and IgA Abs, as well as anti-N IgG Abs [57].

Despite the similarities in the clinical presentation of multisystem inflammatory syndrome in children and Kawasaki disease, these two entities differ in biochemical manifestations, like IL-17A-mediated hyperinflammation in vasculitic disease, but not MIS-C [58]. These findings suggest a different endothelial involvement and immunopathology in these two syndromes. MIS-C patients also had a reduced neutralizing activity compared with other COVID-19 patients, probably caused by a reduced protective serological response [57].

## 7. Vaccination

There is currently no relevant evidence on the safety of Sars-Cov-2 vaccination in children under sixteen/eighteen years of age because the studies necessary for the marketing of the various vaccines did not include, at least in this first phase, the pediatric population or pregnant women. The debate is also open on immunosuppressed patients (children but also adults), who do not seem to manifest more severe forms than the general population [59]. Vaccine strategies for COVID-19, as for some other respiratory viral infections, also require additional safety considerations related to the possibility of antibody-dependent enhancement of the disease [60]. Several scientific societies, such as the Italian Society of Neonatology, have come out in favor of vaccinating pregnant and breastfeeding women [61], while there are no official recommendations for children, although alternative strategies that indirectly focus on protecting children by providing immunization of key categories strictly related to childhood (e.g., doctors, teachers, and grandparents) have been suggested [62].

## 8. Conclusions

Sars-Cov-2 disease, in the current state of knowledge, often has a mild severity in pediatric age patients. The immune system, especially the innate one, partly explains the lower susceptibility of children to this new pathology; despite this, immune dysregulation can lead to severe forms of COVID-19, even in pediatric patients. To even more complicated the understanding of COVID-19 in the pediatric population even more complicated, recent studies have described the presence of long COVID in children, a scenario possibly characterized by a specific long-lasting inflammatory/immunological dysregulation that still needs to be understood [63]. Future studies should aim to investigate the relationship between the underlying immune profile and susceptibility to infection, disease severity, and development of long COVID, in order to highlight which children are most at risk of contracting the virus and experiencing disease progression, with worsening clinical outcomes, and personalize the management.

## Figures and Tables

**Table 1:** Summary of the main immune responses to severe acute respiratory syndrome coronavirus 2 (SARS-CoV-2) in children.

Immune System	Type of Responses
Skin and mucous membranes	IgA response in secretions; ACE2 receptor-mediated virus adhesion, and expressed less in children
Complement system	Activated by lectin pathway; inflammatory response through mediators (C3a and C5a)
Cytokines	Cytokine storm with increased IL-6, IL-2, IL-7, IL-10, and G-CSF
Mast cells	Increase in activity and histamine production
Monocytes and macrophages	Increase in number and activity
White blood cells	Normal WBC in most patients; rare lymphocytosis; lymphopenia (with low eosinophils) is the major finding in the case of altered white blood cells count
